# Current Status of New Anticoagulants in the Management of Venous Thromboembolism

**DOI:** 10.1155/2012/856341

**Published:** 2012-03-08

**Authors:** Roberto C. Montoya, Ajeet Gajra

**Affiliations:** Division of Hematology-Oncology, Department of Medicine, SUNY Upstate Medical University, 750 E Adams St., Syracuse, NY 13210, USA

## Abstract

Venous Thromboembolism, manifested as deep venous thrombosis and pulmonary embolism, is a common problem associated with significant morbidity, mortality, and resource expenditure. Unfractionated heparin, low-molecular-weight heparin, and vitamin K antagonists are the most common treatment and prophylaxis, and have demonstrated their efficacy in a vast number of previous studies. Despite their broad use, these agents have important limitations that have led to the development of new drugs in a bid to overcome the disadvantages of the old ones without decreasing their therapeutic effect. These novel medications, some approved and others in different stages of development, include direct thrombin inhibitors like dabigatran etexilate, and direct activated factor X inhibitors like rivaroxaban. The current paper will review the characteristics, clinical trial results, and current and potential therapeutic uses of these new agents with a focus on the categories of direct thrombin inhibitors and activated factor X inhibitors.

## Introduction

Venous thromboembolism (VTE) is a disease that encompasses the diagnosis of deep vein thrombosis (DVT) and pulmonary embolism (PE). Despite being a preventable problem, VTE has a high prevalence. Without prophylaxis, the incidence of hospital-acquired DVT is approximately 10% to 40% among medical or general surgical patients and 40% to 60% following major orthopedic surgery. Also, approximately 10% of hospital deaths are caused by PE [1]. The effectiveness of primary thromboprophylaxis, to reduce the frequency of DVT and PE, is supported by well-established scientific evidence. Heparin products that include unfractionated heparin (UH), low-molecular-weight heparin (LMWH), and vitamin K antagonists (VKA) are the most commonly used prophylactic treatments and they have demonstrated good efficacy and cost effectiveness. While these agents have been used for many years, each class has its drawbacks and are far from being “ideal” anticoagulants. For this reason, the search for new anticoagulants continues and these efforts have been concentrated on drugs focusing on two targets: thrombin and activated factor X (FXa). 

These novel agents, currently approved or under evaluation for management of VTE, act directly on the active sites of thrombin or FXa and they include the direct thrombin inhibitor (DTI) Dabigatran Etexilate and the direct FXa inhibitors: rivaroxaban, apixaban, edoxaban, and betrixaban.

## 1. Direct Thrombin Inhibitors

DTIs are agents that neutralize thrombin directly by binding to its active catalytic site and blocking its interactions with its substrates. Thrombin plays a central role in the clotting process. As a point of convergence of the two pathways of the coagulation cascade, thrombin converts soluble fibrinogen to fibrin and activates factors V, VIII, and XI which generate more thrombin. It also stimulates platelets and stabilizes the clot by activating factor XIII which favors the formation of cross-linked bonds among the fibrin molecules [2]. DTIs include the parenteral drugs argatroban, bivalirudin, hirudin, and the only oral DTI available dabigatran etexilate, which has been developed most recently.

### 1.1. Dabigatran Etexilate

Dabigatran etexilate (DE) is an orally administrated, specific, and potent reversible thrombin inhibitor. It is a prodrug that is rapidly transformed into its active metabolite dabigatran by a mechanism independent of the CYP enzymes and other oxidoreductases. DE reaches maximal plasma concentrations within two hours of administration [3] or within four hours if it is given with food. This variability has no final effect in the action of the drug [4]. Dabigatran etexilate exhibits linear pharmacokinetic characteristics as reported in a previous study in healthy volunteers and has a percentage of binding to plasma proteins of about 35%. Dabigatran clearance is predominantly renal, with 80% excreted unchanged in the urine and for this reason needs a dose adjustment when administered to subjects with a creatinine clearance <50 mL/min [4]. DE prolongs in a dose-dependent fashion some coagulation tests, including activated partial thromboplastin time (aPTT), thrombin time, and ecarin clotting time. Although aPTT correlates with plasma concentration time profile of dabigatran, this test is not suitable for precise quantification of its anticoagulant effect. On the other side, the effect of dabigatran on the prothrombin time (PT) is minimal at therapeutic doses [5]. Currently, there is no antidote to reverse the antithrombotic effect of dabigatran; however, factor VIIa is a potential candidate since it has shown its ability to reverse the prolonged bleeding time in rats treated with high doses of dabigatran [4].

#### 1.1.1. Clinical Trials of Dabigatran in VTE

In 2008, DE was approved in Europe as a primary prevention of venous thromboembolic events in adult patients who have undergone elective total hip replacement or total knee replacement surgery. In October 2010, DE was FDA approved to reduce the risk of stroke and systemic embolism in patients with nonvalvular atrial fibrillation. Currently DE is not indicated in the USA for any VTE event; however there are ongoing clinical trials evaluating this potential indication and more, under the REVOLUTION trial program which encompasses all the studies described below.

**Primary Prevention Trials**

(a) RE-MODEL is a phase III clinical trial, conducted mainly in Europe, that compared enoxaparin 40 mg SQ once daily (first dose given in the evening before surgery) with DE 150 mg and 220 mg once daily (started 4 hours post operatively), for prevention of VTE after an elective total knee replacement (TKR). The duration of treatment was 6–10 days. The incidence of VTE was 36.4% and 40.5% for 220 mg and 150 mg doses, respectively, and 37.7% for enoxaparin. The safety profile was similar for the three groups. These results showed that both doses of dabigatran were noninferior to enoxaparin [6].

(b) RENOVATE is a phase III clinical trial, conducted mainly in Europe, that compared enoxaparin 40 mg SQ once daily (first dose given in the evening before surgery) with DE 150 mg and 220 mg once daily, for the prevention of VTE after an elective total hip replacement (THR). The duration of the treatment was 28–35 days. The incidence of VTE was 6% and 8.6% for 220 mg and 150 mg doses, respectively, and 6.7% for enoxaparin. The incidence of major bleeding was not significantly different among the three groups. The results showed that either dose of DE was noninferior to enoxaparin [7].

(c) RENOVATE II is a phase III clinical trial that compared enoxaparin 40 mg SQ once daily (first dose in the evening before surgery) with DE 220 mg once daily (starting 1–4 hours after surgery) for the prevention of VTE after THR, during a period of treatment of 28–35 days. RENOVATE II is similar to RENOVATE and aims to further evaluate the efficacy and safety of DE 220 mg dose in a more diverse population, including patients from North America. The results showed that DE was as effective as enoxaparin (7.7% versus 8.8% with a *P* < 0.0001 for the pre-specified non-inferiority margin) for preventing VTE and death from all causes and superior to enoxaparin for reducing the risk of major VTE (proximal DVT and/or PE). The incidence of major bleeding and adverse effects was similar between both groups [8].

(d) REMOBILIZE is a phase III study, conducted mainly in USA and Canada, that compared enoxaparin 30 mg SQ twice daily (starting 12–24 h post surgery) with DE 150 mg and 220 mg once daily, for prevention of VTE after an elective TKR. The duration of treatment was 12–15 days. The incidence of VTE was 31.1% (*P* = 0.02 versus enoxaparin) and 33.7% (*P* < 0.001 versus enoxaparin) for 220 mg and 150 mg doses, respectively, and 25.3% for enoxaparin. This trial demonstrated that dabigatran was inferior to enoxaparin; however the safety profile was equivalent [9].

**Treatment Trials**

(a) RECOVER is a phase III clinical trial that evaluated the use of DE for 6-month treatment of acute symptomatic VTE, as a replacement for VKAs. It compared dabigatran 150 mg twice daily with dose-adjusted warfarin to achieve an INR of 2-3 preceded by initial treatment for 5–10 days with parenteral anticoagulation. The results showed that dabigatran was noninferior to warfarin (2.4% versus 2.1% with a *P* < 0.001) in preventing recurrent VTE; major bleeding events were comparable between both drugs and for any bleeding events dabigatran showed a significant 29% reduction (*P* = 0.0002) in comparison to warfarin [10].

(b) RECOVER 2 is a currently ongoing clinical trial similar to RECOVER. It evaluates DE 150 mg twice daily compared to warfarin (INR 2.0-3.0) for 6-month treatment of acute symptomatic VTE, after initial treatment (5–10 days) with a parenteral anticoagulant. This trial aims to demonstrate the safety and efficacy of DE for the long-term treatment and secondary prevention of VTE [11].

(c) REMEDY is a phase III clinical trial designed to measure the efficacy and safety of DE as a treatment of VTE for an extended period of time. In this study, patients were randomized to receive DE 150 mg BID, administered orally or warfarin (INR 2-3) for 6 to 36 months, after being treated with standard doses of an approved anticoagulant for 3 to 12 months for confirmed acute symptomatic VTE. The results showed that DE was as effective as warfarin (1.8% versus 1.3% with a *P* = 0.03) to prevent recurrent VTE during the extended period of treatment and also was associated with a reduced risk for bleeding in comparison to warfarin. On the other hand, there was a significant increased incidence of acute coronary events in the group that received DE (0.9% versus 0.2% with a *P* = 0.02) [12].

(d) RESONATE is a phase III clinical trial that, like REMEDY, evaluates the use of dabigatran as treatment of VTE for an extended period of time. In this trial, DE 150 mg BID was compared to placebo in the long-term prevention of VTE in patients who completed 6–18 months of treatment with a vitamin K antagonist. After an intervention period of 6 months, recurrent VTE occurred in 0.4% and 5.6% of patients treated with DE and placebo, respectively, which constitutes a 92% relative risk reduction for recurrent VTE. Clinically, relevant bleeding occurred more frequently in the group treated with DE (5.3% versus 1.8% with a *P* = 0.001); however there was not significant difference in the incidence of major bleeding between both groups [13].

## 2. Direct Activated Factor X Inhibitors

Activated factor X in interaction with activated factor V is responsible for the conversion of prothrombin to thrombin. The capacity of one molecule of FXa to generate 1000 molecules of thrombin [14] is well-exploited by the direct FXa inhibitors to lessen the production of thrombin which is responsible of converting fibrinogen to fibrin and activating platelets and factors V, VIII, and XI. The final effect of the decreased thrombin levels is the interruption of the clot formation. In general, direct FXa inhibitors have a broad therapeutic window, low patient variability, and minimal drug or food interactions. For these reasons, like dabigatran, they don't need routine laboratory monitoring [15]. 

The agents in this class that are furthest along in clinical testing include rivaroxaban, apixaban, edoxaban, and betrixaban.

### 2.1. Rivaroxaban

Rivaroxaban is a direct FXa inhibitor, already approved in Europe for the prevention of VTE after THR and TKR. Rivaroxaban is a very specific inhibitor of the FXa and, in contrast to the indirect FXa inhibitor fondaparinux, it is able to inactivate free and clot-associated FXa as well as prothrombinase activity. Rivaroxaban is administered orally once a day, has a bioavailability of about 80% [16], and after being rapidly absorbed reaches the Cmax 2–4 hours after. In plasma, >90% of rivaroxaban is found bound to plasma protein and has half life of up to 12-13 hours in healthy elderly subjects [17]. One-third of the drug is eliminated unchanged in the urine and the other two-thirds are metabolized in the liver via CYP3A4, CYP2C8, and CYP-independent mechanisms with part of the metabolites excreted in the feces and other part eliminated in the urine. Due to its mechanisms of elimination, rivaroxaban is contraindicated in patients with a CLCr <30 mL/min and should be administrated with caution in patients with renal and hepatic insufficiency. The use of rivaroxaban in conjunction with azoles, ritonavir, and other potent CYP3A4 and P-gp inhibitors could interfere with its metabolism and should be avoided. Rivaroxaban dose-dependent inhibition of the FXa prolongs the PT and APTT. This effect on both tests is short lived only and not appropriate to monitor the drug activity. PT is prolonged longer if rivaroxaban is co administrated with food [18].

#### 2.1.1. Clinical Trials of Rivaroxaban in VTE

Rivaroxaban was approved in Europe and many other countries based on the results of the RECORD (Regulation of Coagulation in Orthopedic surgery to prevent DVT and PE) phase III clinical trial program, which enrolled more than 12500 patients. Other studies have been developed also for prophylaxis and treatment of VTE.

**Primary Prevention Trials**

(a) RECORD1 compared rivaroxaban 10 mg daily, 6–8 h post elective THR versus enoxaparin 40 mg daily, 12 h preoperatively. The duration of the treatment was 34 days. Rivaroxaban was significantly superior to enoxaparin for the prevention of VTE and all-cause mortality (1.1% versus 3.7%, *P* < 0.001) without a significant difference in the rates of major bleeding or clinically relevant non-major bleeding [19].

(b) RECORD2 compared rivaroxaban 10 mg daily, 6–8 h after elective THR, versus enoxaparin 40 mg daily, started 12 h preoperatively. The duration of treatment was 31-to-39-day course of rivaroxaban versus 10-to-14-day course of enoxaparin followed by 21 to 25 days of placebo. Rivaroxaban demonstrated superiority over enoxaparin for the primary outcome of total VTE and all-cause mortality (2.0% versus 9.3%, *P* < 0.0001). There was no significant difference in the rates of bleeding between both treatments [20].

(c) RECORD3 compared rivaroxaban 10 mg daily, 6–8 hours after TKR, with enoxaparin 40 mg daily, started 12 h preoperatively, for 10 to 14 days (short-term prophylaxis). This study demonstrated that rivaroxaban was superior to enoxaparin for the prevention of a composite of VTE and all-cause mortality (9.6% versus 18.9%, *P* < 0.001). There was no significant difference in the rates of bleeding between both treatments [21].

(d) RECORD4 compared the efficacy and safety of rivaroxaban 10 mg PO daily, 6–8 hours after elective TKR with enoxaparin 30 mg SQ BID, started 12 h preoperatively. The duration of treatment was 10–14 days. The results demonstrated significant superiority for rivaroxaban over enoxaparin for the primary efficacy endpoint, a composite of total VTE and all-cause mortality (6.9% versus 10.1%, *P* = 0.0118). There was no significant difference in the rate of major bleeding between both regimens [22].

(e) MAGELLAN is a phase III clinical trial that compared the efficacy of rivaroxaban 10 mg PO daily for 35 days versus the efficacy of standard 10-day treatment with enoxaparin 40 mg SQ daily to prevent VTE in acutely ill-medical patients. Participants had an average age of 71 years and one or more acute medical conditions, including active cancer, infectious diseases, heart failure, inflammatory/rheumatic diseases, and so forth. For the primary efficacy endpoint, a composite of VTE, and death, at day 10 results showed that rivaroxaban was noninferior to enoxaparin (2.7% versus 2.7% with a *P* = 0.0025 for non-inferiority). At day 35, rivaroxaban was superior to enoxaparin (4.4% versus 5.7% with a *P* = 0.02). Bleeding rates at both 10 and 35 days were higher with rivaroxaban and because of this the net clinical benefit (combination of the efficacy and bleeding endpoints) favored enoxaparin. Since patients in Magellan constituted a heterogeneous group affected by different diseases, a subgroup analysis is currently ongoing to identify patients who could be associated with a net clinical benefit [23].

**Treatment Trials**

(a) EINSTEIN-DVT EVALUATION is a phase III clinical trial comparing rivaroxaban, 15 mg PO BID for 3 weeks followed by 20 mg daily, versus enoxaparin followed by VKA, for 3 to 12 months, in patients with acute symptomatic DVT (no PE). The results showed that rivaroxaban had noninferior efficacy with respect to the primary outcome that was the prevention of symptomatic recurrent DVT (2.1% versus 3.0%, *P* < 0.001). The rate of bleeding was similar between both groups [24].

(b) EINSTEIN PE is a phase III clinical trial, completed but not published yet, that compares rivaroxaban 15 mg BID for 3 weeks followed by 20 mg daily to enoxaparin 40 mg SQ BID for at least 5 days, in combination with VKA (INR 2-3) in the treatment of patients with acute symptomatic PE with or without symptomatic DVT. The primary endpoint is the composite of recurrent DVT and/or PE occurring during the 3-, 6-, and 12-month study treatment periods [25].

(c) EINSTEIN-EXTENSION study is a phase III clinical trial designed to assess the efficacy and safety of rivaroxaban 20 mg daily for 6 to 12 months, versus placebo in patients who had completed 6 to 12 months of anticoagulant treatment for their acute episode of VTE. The incidence of VTE was 1.3% versus 7.1% for rivaroxaban and placebo, respectively (*P* < 0.001). The results demonstrated that rivaroxaban was associated to an 82% relative risk reduction in the recurrence of VTE in this group of patients. The rate of bleeding for the rivaroxaban group was low and non-statistically significant [26].

### 2.2. Apixaban

Apixaban is another oral, potent, reversible, and direct FXa inhibitor that has been tested for VTE treatment and prophylaxis. It is a very selective drug and like rivaroxaban can inhibit free FXa as well as prothrombinase activity. Apixaban has a high oral bioavailability and after a rapid oral absorption in the stomach and small intestine, reaches a Cmax approximately 1–3 hours after administration. Its half-life is 8–15 hours and about 87% is bound to plasma proteins [27]. Apixaban has a multimodal mechanism of elimination. Most of the drug is excreted in the feces, other part via CYP3A4-dependent mechanisms in the liver, and one-fourth of the drug is eliminated in the urine [27]. For this reason apixaban probably could be safely used in patients with renal and hepatic insufficiency; but like rivaroxaban, its concomitant use with potent CYP3A4 inhibitors like ketoconazole and ritonavir, should be avoided. The PT and aPTT are prolonged by the use of apixaban in a concentration-dependent fashion. However; because at therapeutic concentrations the impact of apixaban on the PT and aPTT is minimal, these tests are not sensitive enough for the monitoring of the drug [28]. In general, if ever needed, an FXa inhibition assay is the best way to monitor the activity of apixaban.

#### 2.2.1. Clinical Trials of Apixaban in VTE

Apixaban is in the process of approval in Europe for prophylaxis after major orthopedic surgery. The ADVANCE 1, 2, and 3 trials are the studies presented to support this indication. Other trials to evaluate apixaban for the prevention of VTE in patients hospitalized or with metastatic cancer are also ongoing.

**Primary Prevention Trials**

(a) ADVANCE-1 is a phase III study that compared apixaban 2.5 mg PO BID with enoxaparin 30 mg SQ BID for prevention of VTE after TKR. Both drugs were started 12–24 h after operation and the duration of treatment was 10–14 days. The results showed that apixaban did not meet the prespecified statistical criteria for non-inferiority (9.0% versus 8.8%), but its use was associated with lower rates of clinically relevant bleeding and it had a similar adverse-event profile [29].

(b) ADVANCE-2 is a phase III clinical trial that compared apixaban 2.5 mg PO BID (12–24 h postoperatively) with enoxaparin 40 mg daily (started 12 h preoperatively) for prevention of VTE after TKR. The results showed that apixaban had noninferior efficacy with respect to the primary outcome that was a composite of total VTE plus all-cause mortality (15% versus 24%, *P* < 0.0001). Further, apixaban was associated with a similar risk of bleeding [30].

(c) ADVANCE-3 is a phase III clinical trial comparing apixaban 2.5 mg PO BID (12–24 h postoperatively) with enoxaparin 40 mg daily (12 h before surgery) for thromboprophylaxis after THR. The primary efficacy outcome, a composite of VTE plus all-cause mortality, occurred in 1.4% of the patients in the apixaban group and in 3.9% of the patients in the enoxaparin group (*P* < 0.0001 for both noninferiority and superiority). The rates of bleeding in both groups were similar. It was concluded that among patients undergoing hip replacement, thromboprophylaxis with apixaban, as compared with enoxaparin, was associated with lower rates of VTE, without increased bleeding [31].

(d) ADOPT is a phase III clinical trial, completed but not published yet, designed to assess the efficacy and safety of apixaban, 2.5 g mg PO BID versus enoxaparin 40 mg SQ daily for prophylaxis of VTE in acutely ill medical subjects during and following hospitalization. The primary efficacy outcome is a composite of VTE and VTE-related death during 30-day treatment [32].

(e) ADVOCATE is a phase II clinical trial, completed but not published yet, designed to know the effectiveness of apixaban as anticoagulant therapy in patients with advanced or metastatic cancer. Patients will be randomized to receive 5 mg daily of apixaban or placebo during 12 weeks. The primary outcome is the occurrence of either a major bleeding (fatal or nonfatal) event or a clinically relevant non-major bleeding event during the treatment period. The secondary outcome is symptoms compatible with VTE [33].

**Treatment Trials**

(a) BOTTICELLI is a phase II clinical trial designed to assess efficacy and safety of 3 different doses of apixaban: 5 mg twice a day, 10 mg twice a day, and 20 mg once daily versus conventional treatment with low-molecular-weight heparin or fondaparinux and vitamin K antagonist in the treatment of subjects with acute symptomatic DVT. The duration of the treatment was three months and the primary efficacy outcome was a composite of symptomatic recurrent VTE and deterioration of thrombotic burden. This study concluded that apixaban can be given as the sole treatment for DVT in a fixed dose and warranted further evaluation of apixaban in phase III studies (AMPLIFY and AMPLIFY-EXT) [34].

(b) AMPLIFY is a phase III study, currently recruiting participants, designed to assess the efficacy and safety of apixaban for the treatment of DVT or PE. It will compare apixaban 10 mg BID for one week followed by 5 mg bid for six months with enoxaparin 1 mg/kg BID followed by warfarin (INR 2-3) for 6 months. The primary outcome is VTE recurrence or death during the study treatment [35].

(c) AMPLIFY-EXT is a phase III study, currently recruiting participants, designed to assess the efficacy and safety of apixaban for extended treatment of DVT or PE. After receiving 6–12 months of treatment for DVT/PE, patients recruited in this study will be randomized to receive apixaban 2.5 mg BID or apixaban 5 mg BID or placebo BID for up to 12 months. The primary outcome is VTE recurrence or death during the study treatment [36].

### 2.3. Edoxaban

Edoxaban is another orally active, reversible and specific inhibitor of the active site of FXa, both free of and within the prothrombinase complex. It has a bioavailability of >50% and after a rapid absorption, in healthy volunteers, it reaches a peak plasma level within 1.5 hours and retains its antithrombotic effect for up to 5 hours after dosing [37]. Edoxaban is eliminated through multiple pathways but predominantly via renal route, so it should be used with caution in patients with renal insufficiency. It has an elimination half-life of 9–11 hours [37]. Edoxaban is metabolized by the P-gp system so its dosage needs to be reduced if is used concomitantly with potent P-gp inhibitors like verapamil and quinidine [37]. Edoxaban prolongs the PT and aPTT in a concentration-dependent fashion, at least in vitro studies [4].

#### 2.3.1. Clinical Trials of Edoxaban in VTE

Edoxaban does not have any indication yet, however; the first trials in Japan have shown that it could be a potential alternative to enoxaparin for prevention of DVT after major orthopedic surgery.

**Primary Prevention Trials**

(a) Fuji et al. [38] in a phase II study evaluated the efficacy and safety of edoxaban for the prevention of VTE in patients undergoing TKR. Patients were randomized to receive edoxaban 5, 15, 30, or 60 mg once daily or placebo for 11–14 days. The incidence of VTE was 29.5%, 26.1%, 12.5%, and 9.1% in the edoxaban 5-, 15-, 30-, and 60-mg treatment groups versus 48.3% in the placebo group. The incidence of bleeding was similar across all the groups. It was concluded that edoxaban demonstrated significant dose-dependent reductions in VTE in patients undergoing TKA with a bleeding incidence similar to placebo.

(b) Raskob et al. [39]: it is a phase II study designed to evaluate the efficacy and safety of different doses of edoxaban for the prevention of VTE in patients undergoing elective THR. Patients were randomized to oral edoxaban 15, 30, 60, or 90 mg once daily or dalteparin SQ once daily (initial dose 2,500 IU, subsequent doses 5,000 IU). Both drugs were begun 6–8 hours postoperatively and continued for 7–10 days. The primary efficacy endpoint was the incidence of total VTE. The incidences of VTE were 28.2%, 21.2%, 15.2%, and 10.6% in patients receiving edoxaban 15, 30, 60, and 90 mg, respectively, compared with 43.8% in the dalteparin group (*P* < 0.005). The incidence of clinically relevant bleeding was low and similar across the groups. It was found that there was a statistically significant (*P* < 0.001) dose-response for efficacy across the edoxaban dose groups for VTE.

(c) STARS J-V is a phase III trial that evaluated the efficacy and safety of edoxaban compared with enoxaparin in patients undergoing THR in Japan. Patients received either 30 mg PO once daily of edoxaban or enoxaparin SQ 20 mg twice daily for 11 to 14 days. The primary efficacy endpoint of the trial was the incidence of PE and DVT. DVT occurred in 2.4% of patients receiving edoxaban compared with 6.9% in the enoxaparin group (*P* = 0.016). There were no PE events observed in either treatment group. There was no statistically significant difference in bleeding episodes. It was concluded that edoxaban demonstrated superior efficacy compared with enoxaparin in preventing VTE after THR [40].

(d) STARS E-3 is a phase III trial that compared edoxaban 30 mg PO daily with enoxaparin 20 mg SQ BID for prevention of VTE in patients undergoing TKR in Japan and Taiwan. The duration of the treatment was 11 to 14 days. The primary efficacy endpoint of the trial was the incidence of PE and DVT. DVT occurred in 7.4% of patients receiving edoxaban and 13.9% of patients who received enoxaparin (*P* = 0.01). No PE was observed in any treatment group. There was no statistically significant difference in the rates of bleeding (*P* = 0.13). It was concluded that Edoxaban was superior to enoxaparin in preventing VTE after TKR [41].

**Treatment Trial**

(a) The Edoxaban Hokusai-VTE study is a phase III clinical trial, currently recruiting participants, designed to evaluate the efficacy and safety of (LMW) heparin/edoxaban versus (LMW) heparin/warfarin in subjects with symptomatic DVT and/or PE. The primary outcome is symptomatic recurrent VTE for 12 months from time of randomization [42].

### 2.4. Betrixaban

Betrixaban is an oral, reversible, and competitive direct FXa inhibitor. Like apixaban and rivaroxaban, betrixaban is a very specific inhibitor of the FXa, both free and bound in the prothrombinase complex [43]. In animal models, betrixaban has a bioavailability of 49% [44]. Its pharmacodynamic half-life is 20 hours and allows an optimal therapeutic range using one daily dose regimen. Elimination is mostly by biliary excretion with minimal renal clearance, which would allow its use in patients with renal insufficiency, without a requirement for dose adjustment. Because of its independence with major CYP P450 enzyme pathways, betrixaban has a minimal potential for drug interactions [43]. 

Betrixaban causes a very minimal prolongation of the PT, aPTT, and the anti-FXa activity [4].

#### 2.4.1. Clinical Trials of Betrixaban on VTE

EXPERT is a phase II clinical trial conducted in the US and Canada that randomized 215 patients undergoing elective TKR to receive betrixaban 15 mg or 40 mg PO BID (starting 6–8 hours after surgery) or enoxaparin 30 mg SQ BID (starting 12 to 24 hours after surgery), for 10–14 days, in order to prevent VTE. The primary efficacy outcome was the incidence of VTE from day 10 to 14. VTE occurred in 20% and 15% of patients receiving betrixaban 15 mg and 40 mg respectively. In the enoxaparin group, 10% of the patients presented VTE. No bleeds were reported for betrixaban 15 mg, two clinically significant nonmajor bleeds (2.4%) with betrixaban 40 mg, and one major (2.3%) and two clinically significant nonmajor (4.6%) bleeds with enoxaparin. The conclusion was that betrixaban demonstrated antithrombotic activity and appeared well tolerated. Further studies are expected to come based on the results of the EXPERT trial [43].

##  Conclusion 

Many new anticoagulants are being currently evaluated for prevention and treatment of VTE. Based on the initial results as outlined above, these agents offer a great promise to be potential substitutes for the current heparin products and VKAs. Also oral route, ease of use, lack of need for routine monitoring, minimal food and drug interactions, and an acceptable safety profile make them attractive. However, they are more expensive and this has raised some questions about the cost effectiveness of these agents. Another concern is the lack of effective antidotes for quick and consistent reversal of anticoagulant effect. As more data emerges, these new agents will find wider applications; although, they are not likely to universally replace heparins and VKAs in the immediate future until the cost and reversal issues are better addressed.
